# Neutrophil-to-Lymphocyte Ratio, Monocyte-to-Lymphocyte Ratio, Platelet-to-Lymphocyte Ratio, and Mean Platelet Volume-to-Platelet Count Ratio as Biomarkers in Critically Ill and Injured Patients: Which Ratio to Choose to Predict Outcome and Nature of Bacteremia?

**DOI:** 10.1155/2018/3758068

**Published:** 2018-07-15

**Authors:** Dragan Djordjevic, Goran Rondovic, Maja Surbatovic, Ivan Stanojevic, Ivo Udovicic, Tamara Andjelic, Snjezana Zeba, Snezana Milosavljevic, Nikola Stankovic, Dzihan Abazovic, Jasna Jevdjic, Danilo Vojvodic

**Affiliations:** ^1^Clinic of Anesthesiology and Intensive Therapy, Military Medical Academy, Crnotravska 17, 11000 Belgrade, Serbia; ^2^Faculty of Medicine, Military Medical Academy, University of Defence, Crnotravska 17, 11000 Belgrade, Serbia; ^3^Institute for Medical Research, Military Medical Academy, Crnotravska 17, 11000 Belgrade, Serbia; ^4^Institute of Medical Biochemistry, Military Medical Academy, Crnotravska 17, 11000 Belgrade, Serbia; ^5^Department of Anesthesiology, Clinical Hospital Center Kosovska Mitrovica, Anri Dinana bb, 38220 Kosovska Mitrovica, Serbia; ^6^Department of Anesthesiology and Intensive Therapy, Mother and Child Health Care Institute of Serbia, Radoja Dakica 8, 11000 Belgrade, Serbia; ^7^School of Medicine, University of Belgrade, Dr Subotica 8, 11000 Belgrade, Serbia; ^8^Emergency Medical Center of Montenegro, Vaka Djurovica bb, 81000 Podgorica, Montenegro; ^9^Clinical Center Kragujevac, Zmaj Jovina 30, 34000 Kragujevac, Serbia; ^10^Faculty of Medical Sciences, University of Kragujevac, Svetozara Markovica 69, 34000 Kragujevac, Serbia

## Abstract

**Background:**

Neutrophil-to-lymphocyte ratio (NLR), monocyte-to-lymphocyte ratio (MLR), platelet-to-lymphocyte ratio (PLR), and mean platelet volume-to-platelet count (MPV/PC) ratio are readily available parameters that might have discriminative power regarding outcome. The aim of our study was to assess prognostic value of these biomarkers regarding outcome in critically ill patients with secondary sepsis and/or trauma.

**Methods:**

A total of 392 critically ill and injured patients, admitted to surgical ICU, were enrolled in a prospective observational study. Leukocyte and platelet counts were recorded upon fulfilling Sepsis-3 criteria and for traumatized Injury Severity Score > 25 points. Patients were divided into four subgroups: peritonitis, pancreatitis, trauma with sepsis, and trauma without sepsis.

**Results:**

NLR and MPV/PC levels were significantly higher in nonsurvivors (AUC/ROC of 0.681 and 0.592, resp., in the peritonitis subgroup; 0.717 and 0.753, resp., in the pancreatitis subgroup); MLR and PLR did not differ significantly. There was no significant difference of investigated biomarkers between survivors and nonsurvivors in trauma patients with and without sepsis except for PLR in the trauma without sepsis subgroup (significantly higher in nonsurvivors, AUC/ROC of 0.719). Independent predictor of lethal outcome was NLR in the whole cohort and in the peritonitis subgroup as well as MPV in the pancreatitis subgroup. Also, there were statistically significant differences in MPV/PC, MLR, and PLR values regarding nature of bacteremia. In general, the lowest levels had been found in patients with Gram-positive blood cultures.

**Conclusions:**

NLR and MPV were very good independent predictors of lethal outcome. For the first time, we demonstrate that nature of bacteremia influences MPV/PC, MLR, and PLR. In heterogeneous cohort subgroup, analysis is essential.

## 1. Introduction

In the treatment of critically ill and/or injured patients, it is important to detect those who are at high risk for lethal outcome. Major determinant of outcome is intensity of insult (infection, trauma) as well as immunoinflammatory response [1, 2]. It is difficult to find adequate biomarker of immune response in critical illness, regardless of its cause, with good predictive value regarding outcome because there is wide and complex array of immune-related mediators. Many of them were explored in this clinical setting with contradictory results. Recently, some readily available parameters, originated from routine complete blood count (CBC), have been investigated as potential biomarkers with mixed results and no consensus so far regarding its accuracy and clinical usefulness: neutrophil-to-lymphocyte ratio (NLR), monocyte-to-lymphocyte ratio (MLR), platelet-to-lymphocyte ratio (PLR), and mean platelet volume-to-platelet count (MPV/PC) ratio [3–6].

Bearing in mind how intertwined immune and coagulation cascades are, the aim of our prospective observational study was to assess the prognostic value of NLR, MLR, PLR, and MPV/PC ratio regarding outcome in a cohort of critically ill patients with secondary sepsis and/or trauma. Outcome measure was hospital mortality. Our secondary endpoint was to assess possible differences of these biomarkers regarding different blood cultures in patients with documented bacteremia.

## 2. Material and Methods

### 2.1. Study Design

A total of 392 critically ill and injured patients, admitted to surgical intensive care unit (SICU), were enrolled in a prospective study conducted in a tertiary university hospital (Military Medical Academy, Belgrade, Serbia) during a 4-year period. Approval in concordance with the Declaration of Helsinki was obtained from local ethics committee as well as informed consent from a patient or first-degree relative. The study was conducted in accordance with the approved guidelines. Patients with secondary sepsis (underlying conditions were peritonitis, pancreatitis, and trauma) were enrolled if they had fulfilled current Sepsis-3 diagnostic criteria for sepsis (formerly severe sepsis) and/or septic shock (acute change in total SOFA score ≥ 2 points and vasopressors required to maintain mean arterial pressure ≥ 65 mmHg and serum lactate level > 2 mmol/L despite adequate volume resuscitation) [7]. The diagnostic criteria encompass any of the following variables thought to be a result of the infection: sepsis-induced hypotension, lactate levels greater than 2 mmol/L, urine output less than 0.5 mL/kg/hr for more than two hours despite adequate fluid resuscitation, acute lung injury with PaO_2_/FiO_2_ less than 250, creatinine greater than 2.0 mg/dL (176.8 micromol/L), bilirubin greater than 2.0 mg/dL (34.2 micromol/L), platelet count less than 100,000, and coagulopathy (international normalised ratio, INR) greater than 1.5. Also, critically ill patients with severe trauma (Injury Severity Score (ISS) (determined using Abbreviated Injury Scale (AIS)) > 25 points) were enrolled. Only adult patients, age of at least 18 years, were recruited. The exclusion criteria were as follows: (1) secondary sepsis and/or septic shock with an underlying condition other than severe peritonitis, pancreatitis, or trauma, (2) malignant disease of any origin, (3) long-term SICU stay before criteria fulfilment, and (4) preexisting immunodeficiency. All trauma patients were further classified into two subgroups: those who developed secondary sepsis (trauma + sepsis subgroup) and those who did not (trauma subgroup). We sought to recruit four homogenous subgroups in a cohort of critically ill and injured patients: peritonitis subgroup, pancreatitis subgroup, trauma + sepsis subgroup (these three subgroups of patients developed secondary sepsis), and trauma subgroup.

### 2.2. Blood Measurement

Patient's venous blood was drawn by trained, qualified phlebotomists. If several measurements were available on day of enrollment in the study for some patients, the first one at the time point of diagnostic criteria fulfillment was always used to maximize consistency among patient population. The blood samples were taken into BD Vacutainer K_2_ EDTA tubes and were analyzed within 2 hours from venepuncture. A complete blood count was determined by the Siemens Advia 120 hematology system, which is a flow cytometry-based system.

Differentiation of white blood cells is done by the peroxidase and basophil channel. The peroxidase method is a primary differential method on the Advia 120. Peroxidase in the granules of the white blood cells reacts with hydrogen peroxide from reagent and forms dark precipitates within the cells. After measuring the light scatter which represents the size of the cell and absorption showing the level of staining, the analyzer defines next cell population: neutrophils, monocytes, eosinophils, and large unstained cells, while lymphocytes and basophils appear as one cluster. This requires a further method of differentiation. The basophil method uses the resistance of basophils to acid lysis and differentiates them from the rest of the white blood cell population. The Advia 120 analyzer method of counting platelets is based on two-dimensional laser light scatter. The laser optics low angle and high angle scatter is used to determine the platelet count simultaneously with the red blood cells. Mean platelet volume (MPV) is a calculated parameter from platelet volume histogram. NLR, MLR, PLR, and MPV/PC were calculated as ratios of circulating neutrophil, monocyte, lymphocyte, and platelet counts, respectively. Normal ranges for these cell counts are as follows: leukocyte 4–10.8 × 10^9^/L; neutrophil 1.9–8 × 10^9^/L; lymphocyte 0.9–5.2 × 10^9^/L; monocyte 0-1 × 10^9^/L; and platelet 130.0–400.0 × 10^9^/L (data from our laboratory).

### 2.3. Disease Severity and Outcome

To assess the severity of secondary sepsis and/or trauma, Acute Physiology and Chronic Health Evaluation (APACHE) II score was performed on admission in all critically ill patients, regardless of underlying condition. Injury Severity Score (ISS) (determined using Abbreviated Injury Scale (AIS)) was calculated and recorded in all trauma patients. Outcome measure was hospital mortality.

### 2.4. Statistical Analysis

In case of continuous data, variables were presented as mean value ± standard deviation (SD) or median followed by interquartile range. Some of the variables were presented as frequency of certain categories, while statistical significance of differences was tested with the chi square test. All variables were tested for normal distribution by Kolmogorov-Smirnov test. In accordance with the result of this test, the statistical significance of differences was tested using the *t*-test or Mann–Whitney *U* test (two groups' comparison). In case of multigroup comparison, ANOVA or Kruskal-Wallis test (post hoc Mann–Whitney test) was applied. The relationship between two variables was established using Spearman correlation analysis (rho value). The determination of cut-off values and sensitivity and specificity of the variables were analyzed using the ROC curve procedure (the Youden index was used in all cases). Calculations of odds ratios and their 95% confidence intervals were done to determine the strength of the association between risk factors and outcomes. For that purpose, the most promising independent variables (univariate analysis) as single risk factors were incorporated into binary logistic regression analyses (multivariate analysis). Differences between groups were considered significant at *p* < 0.05. Complete statistical analysis of the data was conducted with the statistical software package SPSS Statistics 18 (Chicago, Illinois, USA).

## 3. Results

### 3.1. Baseline Characteristics of the Study Population

During a 4-year study period, 392 critically ill and injured patients were enrolled (60.2% male; mean age 53.67 ± 18.26 years). Nonsurvivors were older (mean ± SD, years): 63.21 ± 15.02 versus survivors 45.66 ± 16.86 (*t* = 10.89; *p* ≤ 0.001). According to diagnosis, they were divided into 4 subgroups: peritonitis (*n* = 196; 50.0%), pancreatitis (*n* = 67; 17.1%), trauma with secondary sepsis (*n* = 83; 21.2%), and trauma without sepsis (*n* = 46; 11.7%). According to blood culture, there were 65 (16.6%) patients with isolated Gram-positive pathogens, 15 (3.8%) with Gram-negative pathogens, 184 (46.9%) with polymicrobial blood culture, and 128 (32.7%) with negative blood culture. Overall hospital mortality was 45.7%; there were 179 nonsurvivors. Acute Physiology and Chronic Health Evaluation (APACHE) II score (mean ± SD) was 22.32 ± 3.7 in all critically ill patients, regardless of underlying condition. Injury Severity Score (ISS) (determined using Abbreviated Injury Scale (AIS)) was calculated and recorded in all trauma patients (mean ± SD): 26.36 ± 5.42. Baseline characteristics of all patients according to outcome are shown in Table 1.

Statistical analysis of all patients revealed that female mortality rate was significantly higher (*p* ≤ 0.001), which was not surprising given that females dominantly suffered from peritonitis (*p* ≤ 0.001). Peritonitis and pancreatitis were significantly associated with lethal outcome (*p* ≤ 0.001). On the other hand, males dominantly were traumatized with the development of secondary sepsis (*p* ≤ 0.001). All patients in the trauma subgroup had negative blood culture; those with trauma and secondary sepsis dominantly had polymicrobial blood culture (*p* ≤ 0.001). Males dominantly had negative blood culture (*p* ≤ 0.001) as expected because they comprised almost 70% of the trauma subgroup.

In Table 2, laboratory characteristics of all patients according to outcome are shown.

Lymphocyte, monocyte, and platelet counts were significantly higher, and MPV was significantly lower in survivors. NLR and MPV/PC levels were higher in nonsurvivors, difference reached high statistical significance. MLR and PLR values did not differ significantly between survivors and nonsurvivors.

In Table 3, baseline and laboratory characteristics of all patients according to blood cultures are shown.

Post hoc Mann–Whitney test revealed that both lymphocyte count and monocyte count were significantly higher in patients with Gram-positive blood culture compared to those with polymicrobial and negative blood cultures. Also, patients with Gram-positive blood culture had significantly lower MPV/PC ratio compared to patients with Gram-negative and polymicrobial blood cultures. When comparing polymicrobial and negative blood culture, we found significantly higher MPV and MPV/PC values as well as significantly lower platelet count in patients with polymicrobial blood culture. Patients with Gram-negative, polymicrobial, and negative blood cultures had significantly higher PLR and MLR values in comparison with those who had Gram-positive blood culture. When comparing polymicrobial and negative blood cultures, we found significantly lower PLR and MLR values in patients with polymicrobial blood culture. So, the highest levels of MLR and PLR had been found in patients with negative blood culture and the lowest in patients with Gram-positive blood culture. NLR values did not differ significantly between these four subgroups of patients according to blood culture.

In Table 4, baseline and laboratory characteristics of all patients according to underlying condition are shown.

Post hoc Mann–Whitney test revealed that platelet count was significantly higher in trauma patients compared to those with other underlying conditions (the lowest count was in the peritonitis subgroup). Patients with peritonitis and pancreatitis had significantly higher MPV values compared to trauma patients with and without secondary sepsis. Monocyte count, as well as MLR value, was significantly lower in patients with peritonitis compared to those with other underlying conditions. In the pancreatitis subgroup, NLR value was significantly higher compared to those with other underlying conditions. PLR and MPV/PC values did not differ significantly between these four subgroups of patients according to underlying condition.

### 3.2. Peritonitis Subgroup

There were 196 patients with peritonitis. Baseline and laboratory characteristics of the peritonitis subgroup are shown in Table 1 in Supplementary file. Statistical analysis of laboratory characteristics of the peritonitis subgroup according to outcome showed the same trend and significant differences as in all 392 patients. Clinical accuracy of baseline biomarkers in predicting lethal outcome in the peritonitis subgroup was investigated. Monocytes, lymphocytes, and platelets lower than cut-off values as well as MPV, MPV/PC, and NLR higher than cut-off values are moderate predictors of lethal outcome in patients with peritonitis (Table 2 in Supplementary file). Discriminative power of MLR and PLR regarding outcome in this subgroup of patients was not significant. Clinical accuracy of baseline biomarkers in predicting negative blood culture in this subgroup was also investigated. MPV and MPV/PC lower than cut-off values as well as platelets, MLR, and PLR higher than cut-off values are moderate predictors of negative blood culture in patients with peritonitis (Table 3 in Supplementary file). Discriminative power of other investigated biomarkers regarding negative blood culture in this subgroup of patients was not significant.

### 3.3. Pancreatitis Subgroup

There were 67 patients with pancreatitis. Baseline and laboratory characteristics of the pancreatitis subgroup are shown in Table 4 in Supplementary file. Lymphocyte count and platelet count were significantly higher in survivors, monocyte count showed the same trend, but it did not reach statistical significance. Similar trend, seen in all 392 patients as well as in the peritonitis subgroup, was evident in the pancreatitis subgroup (MPV, MPV/PC, and NLR levels were significantly higher in nonsurvivors; MLR and PLR values did not differ significantly between survivors and nonsurvivors). But, unlike the whole group and the peritonitis subgroup, in the pancreatitis subgroup of patients, there were significantly higher WBC and neutrophil counts in nonsurvivors. Clinical accuracy of baseline biomarkers in predicting lethal outcome in the pancreatitis subgroup was investigated. Lymphocytes and platelets lower than cut-off values as well as MPV, MPV/PC, and NLR higher than cut-off values are very good predictors of lethal outcome in patients with pancreatitis (Table 5 in Supplementary file). Discriminative power of monocyte, MLR, and PLR regarding outcome in this subgroup of patients was not significant. Clinical accuracy of baseline biomarkers in predicting negative blood culture in this subgroup was also investigated. Lymphocyte and platelet counts higher than cut-off values are moderate predictors whereas MPV, MPV/PC, and NLR lower than cut-off values are very good predictors of negative blood culture in patients with pancreatitis (Table 6 in Supplementary file). Discriminative power of other investigated biomarkers regarding negative blood culture in this group of patients was not significant.

### 3.4. Trauma Patients with Secondary Sepsis

There were 83 trauma patients who developed secondary sepsis. Baseline and laboratory characteristics of the trauma + sepsis subgroup are shown in Table 7 in Supplementary file. Unlike patients with peritonitis and pancreatitis, there was no significant difference of investigated laboratory parameters between survivors and nonsurvivors in trauma patients who developed secondary sepsis. Therefore, in this subgroup, none of the investigated biomarkers had significant discriminative power regarding outcome. Clinical accuracy of baseline biomarkers in predicting polymicrobial blood culture in trauma patients with secondary sepsis was investigated. Lymphocyte count lower than cut-off value and NLR higher than cut-off value are moderate predictors of polymicrobial blood culture in trauma patients with secondary sepsis (Table 8 in Supplementary file). Discriminative power of other investigated biomarkers regarding polymicrobial blood culture in this subgroup of patients was not significant.

### 3.5. Trauma Subgroup

There were 46 trauma patients. Baseline and laboratory characteristics of the trauma subgroup are shown in Table 9 in Supplementary file. Unlike all other subgroups of patients, in trauma patients, PLR value was significantly higher in nonsurvivors. Also, in this subgroup, all other laboratory parameters showed no significant difference between survivors and nonsurvivors. Clinical accuracy of baseline biomarker PLR in predicting lethal outcome in the trauma subgroup was investigated. PLR higher than cut-off value is a very good predictor of lethal outcome in trauma patients (Table 10 in Supplementary file). Discriminative power of all other biomarkers regarding outcome in this subgroup of patients was not significant.

### 3.6. Combination of MPV/PC, NLR, MLR, and PLR in a Composite Bioscore

In order to determine whether combination of these four ratios into a one composite bioscore is going to improve their prognostic performance regarding lethal outcome in all subgroups of patients, individual values were scored as 1 or 0 based on their relation (below or above) to previously established ROC curve cut-off levels. Bioscore ranges from 0 to 4 points.

In the peritonitis subgroup, AUC/ROC for composite bioscore was 0.718 (95% confidence interval 0.644–0.792; *p* ≤ 0.001). This is shown in Figure 1B in Supplementary file. Composite bioscore of 1 point had a sensitivity of 63.5% and a specificity of 68.0% (Youden index = 0.31). Bioscore higher than this cut-off value is a good predictor of lethal outcome. In Figure 1B in Supplementary file, percentage of nonsurvivors for each bioscore point value is shown.

**Figure 1:**
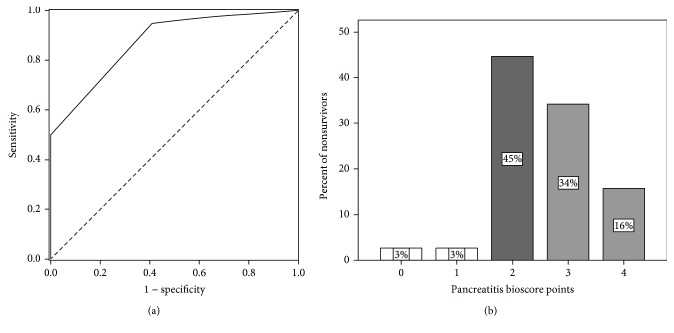
(a) Receiver operating characteristic (ROC) curve for composite bioscore in the pancreatitis subgroup and the lethal outcome (AUC = 0.874). (b) Percentage of nonsurvivors according to each bioscore point value in the pancreatitis subgroup (darkest shade: cut-off bioscore point value).

In the pancreatitis subgroup, AUC/ROC for composite bioscore was 0.874 (95% confidence interval 0.791–0.956; *p* ≤ 0.001). This is shown in Figure 1(a). Composite bioscore of 2 points had a sensitivity of 72.5% and a specificity of 79.5% (Youden index = 0.51). Bioscore higher than this cut-off value is a very good predictor of lethal outcome. In Figure 1(b), percentage of nonsurvivors for each bioscore point value is shown.

In the trauma with secondary sepsis subgroup, AUC/ROC for composite bioscore was 0.782 (95% confidence interval 0.677–0.888; *p* ≤ 0.001). This is shown in Figure 2A in Supplementary file. Composite bioscore of 2 points had a sensitivity of 71.1% and a specificity of 68.2% (Youden index = 0.39). Bioscore higher than this cut-off value is a good predictor of lethal outcome. In Figure 2B in Supplementary file, percentage of nonsurvivors for each bioscore point value is shown.

In the trauma subgroup, AUC/ROC for composite bioscore was 0.823 (95% confidence interval 0.673–0.974; *p* = 0.001). This is shown in Figure 3B in Supplementary file. Composite bioscore of 3 points had a sensitivity of 50.0% and a specificity of 94.3% (Youden index = 0.44). Bioscore higher than this cut-off value is a good predictor of lethal outcome. In Figure 3B in Supplementary file, percentage of nonsurvivors for each bioscore point value is shown.

### 3.7. Correlation between MPV/PC, NLR, MLR, and PLR

**Figure 2:**
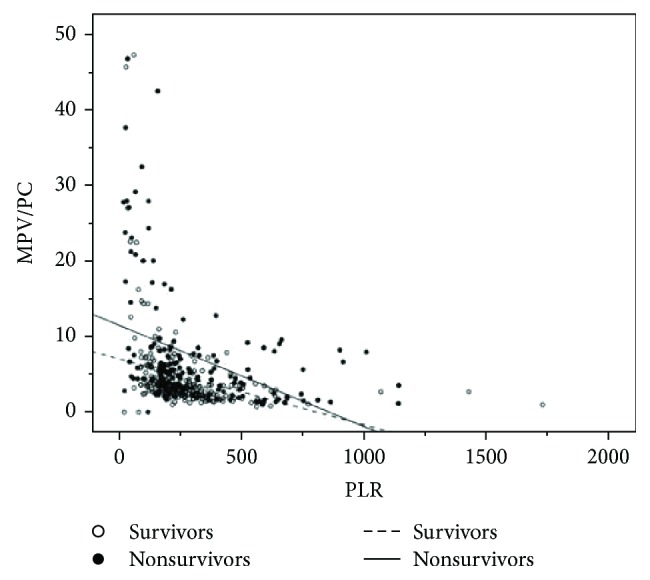
Scattergram of MPV/PC versus PLR for all patients (survivors and nonsurvivors).

A Spearman rho test of correlation between MPV/PC, NLR, MLR, and PLR was performed. In general, regarding all patients, there were significantly positive correlations between investigated biomarkers, regardless of outcome (Table 11 in Supplementary file). In both survivors and nonsurvivors, there was a significantly positive correlation between PLR and NLR or MLR; there was a significantly negative correlation between PLR and MPV/PC (Figure 2).

Also, there was a significantly positive correlation between MLR and NLR. The correlation between MPV/PC and NLR or MLR was not significant.

In the peritonitis subgroup, there was exactly the same trend of correlations between same biomarkers, with same levels of significance.

In the pancreatitis subgroup, the same trend of correlations with same biomarkers and levels of significance continued with one addition: there was a significantly negative correlation between MLR and MPV/PC in nonsurvivors (rho = −0.418; *p* = 0.009).

In trauma with secondary sepsis nonsurvivors, the same trend of correlations with same biomarkers and levels of significance continued. But, in survivors from this subgroup, trend was slightly different: correlation between PLR and MLR was not significant.

In the trauma subgroup, there was a different trend of correlations in both survivors and nonsurvivors (Table 12, Figure 4 in Supplementary file). In both survivors and nonsurvivors, there was a significantly negative correlation between PLR and MPV/PC; also, there was a significantly negative correlation between PLR and MLR in nonsurvivors and a significantly positive correlation between same two biomarkers in survivors. Also, there was a significantly positive correlation between MLR and MPV/PC in nonsurvivors as well as between MLR and NLR in survivors. Other correlations were not significant.

In survivors, regardless of underlying condition, there was exactly the same trend of correlations between same biomarkers, with same levels of significance in patients with Gram-positive and negative blood cultures (Table 13 in Supplementary file).

In survivors with Gram-positive and negative blood cultures, there was a significantly positive correlation between PLR and NLR or MLR; there was a significantly negative correlation between PLR and MPV/PC. Also, there was a significantly positive correlation between MLR and NLR. The correlation between MPV/PC and NLR or MLR was not significant.

In survivors with polymicrobial blood cultures, there was a significantly negative correlation between PLR and MPV/PC (rho = −0.566; *p* ≤ 0.001) as well as a significantly positive correlation between PLR and NLR (rho = 0.310; *p* = 0.024). Other correlations were not significant.

In survivors with Gram-negative blood cultures, the only significant correlation was positive one between NLR and MPV/PC (rho = 0.618; *p* = 0.030).

There were no significant correlations between investigated biomarkers in nonsurvivors with Gram-negative blood cultures.

In nonsurvivors with Gram-positive and polymicrobial blood cultures, there was a similar trend in correlations between investigated biomarkers (Table 14 in Supplementary file).

In nonsurvivors with negative blood cultures, there were only two significant correlations between MPV/PC on one hand and NLR (positive correlation; rho = 0.590; *p* = 0.001) and PLR (negative correlation; rho = −0.633; *p* ≤ 0.001) on the other hand.

### 3.8. Independent Prognostic Significance of Cells and Ratios in Predicting Lethal Outcome

Univariate logistic regression analyses were performed in order to determine whether associations of each individual variable with lethal outcome exist. Standardized regression coefficient (*β*) and the odds ratio (OR) with 95% CI were calculated for each variable. Forward stepwise multivariate logistic regression model was performed in order to determine the independent predictors of lethal outcome, without the effect of possible confounders. In Table 5, univariate odds ratios of variables for predicting lethal outcome in all patients are shown.

Independent predictors of lethal outcome by multivariate logistic regression analysis in all patients are shown in Table 6.

Among categorical variables, the most important independent predictor of lethal outcome is polymicrobial blood culture, followed by female gender and pancreatitis as underlying condition, respectively. As far as continuous variables are concerned, the most important independent predictor of lethal outcome is older age, then comes higher NLR and lower platelet count, respectively. Multivariate logistic regression analysis demonstrated that polymicrobial blood culture remained statistically highly significant independent predictor of lethal outcome when compared to both Gram-positive (standard *β* value 3.950; OR 51.922, 95% CI 13.777–195.681; *p* ≤ 0.001) and Gram-negative (standard *β* value 3.117; OR 22.584, 95% CI 4.465–114.231; *p* ≤ 0.001) blood cultures.

Univariate and multivariate logistic regression analyses were performed in the same fashion in the peritonitis subgroup. Here again, the peritonitis subgroup showed very similar trend compared to the whole cohort of critically ill and injured patients (Tables 15 and 16 in Supplementary file). Among categorical variables, the most important independent predictor of lethal outcome is polymicrobial blood culture, followed by female gender. As far as continuous variables are concerned, the most important independent predictor of lethal outcome is older age, then comes higher NLR value. Multivariate logistic regression analysis demonstrated that polymicrobial blood culture remained statistically highly significant independent predictor of lethal outcome when compared to both Gram-positive (standard *β* value 3.539; OR 34.446, 95% CI 7.422–159.874; *p* ≤ 0.001) and Gram-negative (standard *β* value 2.710; OR 15.034, 95% CI 2.633–85.828; *p* = 0.002) blood cultures.

Slightly different trend was demonstrated in the pancreatitis subgroup regarding univariate and multivariate logistic regression analyses of lethal outcome predictors (Tables 17 and 18 in Supplementary file). As far as continuous variables are concerned, the most important independent predictor of lethal outcome is higher MVP value, then older age and lower lymphocyte count, respectively.

In patients with and without secondary trauma, univariate logistic regression analyses demonstrated that only older age is the significant independent predictor of lethal outcome (trauma with secondary sepsis subgroup: standard *β* value 0.075; OR 1.078, 95% CI 1.036–1.121; *p* ≤ 0.001; trauma subgroup: standard *β* value 0.222; OR 1.249, 95% CI 1.063–1.467; *p* = 0.007).

## 4. Discussion

Our prospective observational study focused on prognostic value of four different cell ratios, NLR, MLR, PLR, and MPV/PC, primarily regarding lethal outcome and secondarily regarding nature of blood cultures in a heterogeneous cohort of critically ill patients with secondary sepsis and/or trauma. We chose these parameters because of their availability in routine clinical practice in a real-life setting. We analyzed our critically ill patient population cohort as a whole, as well as divided it into four subgroups according to underlying condition separately, due to heterogeneity.

The key cell types of the innate immune system as well as the first cellular line of defense against infection are neutrophils. Lymphocytes are involved in adaptive immune response. Immune response to various insults frequently has distinctive feature: increase in neutrophil count and decrease in lymphocyte count. When infection persists, large amount of neutrophils is produced and they might not become apoptotic. Apoptosis of neutrophils in sepsis is beneficial, in contrast to lymphocytes. In this study, circulating cell counts were investigated. Next, logical step would be adding phenotypic markers into analysis. But, swift implementation of these markers is hampered by costs and accessibility.

In a study regarding NLR as predictor of lethal outcome in sepsis, Liu and coworkers [3] reported results similar to ours. NLR was significantly higher in nonsurvivors, with slightly higher AUC than in our study, and in our investigation, NLR was an independent predictor of lethal outcome. In that study, other ratios were not explored, and unlike our results, they reported no significant difference in platelet count between survivors and nonsurvivors. Also, their outcome measure was 28-day mortality and ours was hospital mortality, which is, in our opinion, a better choice. Either way, choice of outcome measure influences results. Association between NLR and mortality in critically ill was investigated by Salciccioli and coworkers in an observational cohort study [8]. They found a statistically significantly higher baseline NLR values in patients with sepsis compared to those without sepsis contrary to our results. Also, opposite to our results, they found the strongest relationship between NLR and mortality in patients without sepsis. In our study, it is the trauma subgroup in which there was no significant difference in NLR values between survivors and nonsurvivors. These authors found an association between NLR and outcome in whole cohort of critically ill patients like we did. In accordance with our results regarding NLR in pancreatitis subgroup are findings reported by Azab and coauthors [9]. They also found that NLR was a good predictor of adverse outcome of acute pancreatitis in ICU setting. In a different population of patients with the first episode of community-acquired or healthcare-related bacteremia during hospital admission, Terradas and coauthors found that persistence of an NLR > 7 was an independent marker of mortality [10]. Trend was the same in our study, only NLR values with the best sensitivity and specificity as predictors of lethal outcome were higher: in the peritonitis subgroup 13.25 and in the pancreatitis subgroup 10.44, which is not surprising given that our patient population was critically ill in ICU setting. Interesting study regarding reversal of NLR in early (in the first week) versus late (within or beyond the first month) death from septic shock was conducted by Riché and coauthors [11]. In the whole cohort of 130 patients, authors found that neutrophil count at admission was similar between survivors and nonsurvivors. Same was true in our study except for the pancreatitis subgroup. They also found that lymphocyte count was higher and NLR reduced in nonsurvivors. These findings are in contrast to those in our study regarding nonsurvivors: lymphocyte count was lower in the whole cohort as well as in all subgroups of our patients and NLR was significantly higher in the whole cohort, the peritonitis subgroup, and the pancreatitis subgroup, while in trauma patients with and without sepsis NLR was lower in nonsurvivors, but it did not reach statistical significance. In the literature available to us, studies regarding cell counts and ratios in general—and NLR specifically in trauma patients—are sparse at best and almost always focused on surgical patients [12]. In a large cohort study on 1356 adult trauma patients (median ISS of 13, comparable to our trauma subgroups), admitted to the surgical ICU of a level 1 trauma center, predictive capacity of NLR on mortality was assessed [13]. Authors performed ROC curve analyses at ICU days 2 and 5, and they found optimal NLR cut-off values of 10.45 and 7.91, respectively. They calculated cut-off values by maximizing the Youden index as we did. Also, in their study, NLR greater than or equal to these cut-off values was a marker for increased hospital mortality. These findings are in contrast to those in our study regarding trauma nonsurvivors; NLR was insignificantly lower in trauma with and without sepsis nonsurvivors, with no significant discriminative power regarding outcome. NLR has good prognostic accuracy regarding outcome, generally higher than traditional infection markers, not only in sepsis but also in a variety of infective states [14].

Several studies investigated diagnostic value of NLR for bacteremia. In emergency department setting, de Jager and coauthors found significant differences between patients with positive and negative blood cultures in lymphocyte count and NLR [15]. Patients with positive cultures had lower lymphocyte count (0.8 versus 1.2 × 10^9^/L) and higher NLR (20.9 versus 13.2); also, AUC/ROC of 0.73 for both biomarkers was high. These results were in accordance with our results. We investigated clinical accuracy of cell counts and ratios in predicting negative blood cultures in the peritonitis subgroup and the pancreatitis subgroup. Higher lymphocyte count (AUC 0.68) and lower NLR (AUC 0.72) were good predictors of negative blood culture in the pancreatitis group; interestingly, their discriminative power in the peritonitis group was not significant. Similar results regarding evaluation of NLR as a diagnostic biomarker for positive versus negative blood cultures were reported by Zhang with coauthors [16]. NLR values were significantly higher in patients with positive blood cultures, diagnostic performance was good (AUC 0.71) as in our pancreatitis subgroup. In these two studies [15, 16], underlying condition leading to sepsis was not reported, in one of them [15] comorbidities were. Our cut-off value for NLR in the pancreatitis subgroup was 13.00; this is in accordance with the proposed appropriate cut-off value of NLR for sepsis (13–15) [17].

Monocytes are an essential component of the innate immune response that acts as a link to the adaptive immune system via antigen presentation to lymphocytes. Literature regarding MLR in infection is scarce. One study investigated NLR and MLR in discriminating between different patient groups hospitalized for fever due to infection and those without infection [4]. They concluded that both NLR and MLR may be useful in the diagnosis of bacterial infection, AUCs 0.708 and 0.688, respectively. Also, authors reported that the highest MLR had been found in patients with confirmed bacterial infection and lower MLR was found in patients with clinically diagnosed infection which was not supported by microbiology. This is in contrast to MLR values in our study: MLR was a moderate predictor of negative blood culture in the peritonitis subgroup with AUC of 0.586 (*p* = 0.046); patients with negative blood culture had significantly higher MLR values. Variability of MLR values was demonstrated in study regarding this cell ratio in patients with active tuberculosis [18]. Authors found that patients with active tuberculosis had a higher or lower MLR compared to controls and that MLR in extreme percentiles were significant predictors of active tuberculosis (<9% or >25%).

MLR was assessed as a predictor of survival in patients with various malignant diseases [19]. In one study, authors concluded that this ratio predicts patient survival and aggressiveness of endometrial cancer [20]. Given that there is an association between inflammation and cancer and that chronic inflammation is important in the malignant transformation, promotion, and metastasis of cancer, we investigated MLR as a predictor of lethal outcome. Differences between MLR values in survivors and nonsurvivors were not significant in neither of our subgroup of patients or in the whole cohort. Univariate and multivariate logistic regression analyses confirmed that MLR has no independent prognostic significance regarding outcome.

Systemic inflammation is an integral part of disease progression in critical illness and is commonly associated with sepsis, leading to an increased risk of mortality. Prognostic value of PLR in critically ill patients with acute kidney injury was assessed by Zheng and coauthors [5]. They observed a U-shaped relationship between PLR and both 90-day and 30-day mortality, with the lowest risk being at values ranging from 90 to 311, so both low and high PLRs were associated with increased mortality. Authors concluded that PLR appears to be a novel, independent prognostic marker of outcome. This is in partial contrast to PLR values in our study except for our trauma subgroup. In traumatized patients, nonsurvivors had significantly higher PLR values. Interestingly, in this subgroup of our patient population, all other laboratory parameters showed no significant difference between survivors and nonsurvivors. PLR higher than cut-off value was a very good predictor of lethal outcome (AUC 0.719; *p* = 0.030). Contrary to our PLR results in the trauma subgroup of critically ill patients, Emektar and coauthors reported that in somewhat different patient population of older patients with hip fractures, PLR values were higher in survivors, but with rather low discriminative power (AUC 0.56) [21]. In our study, PLR values were highest in patients with negative blood culture in the whole cohort of critically ill patients, and it was statistically highly significant. PLR also had moderate discriminative power in predicting negative blood culture in the peritonitis subgroup (AUC 0.613; *p* = 0.020). In accordance with our results are findings from Bekdas and Ozturk, who, in different clinical setting but also related to infection, assessed diagnostic accuracy of PLR regarding acute complicated appendicitis (defined as a the presence of phlegmon, abscess, or perforation) in pediatric population [22]. In their study, in diagnosis of complicated acute appendicitis, PLR had a sensitivity of 62.5% and a specificity of 61.8%. So, PLR, as a novel biomarker, has been investigated recently in various clinical settings [23, 24].

Sepsis is associated with hemostatic system dysfunction, and platelets play important role in both hemostasis and immunoinflammatory response to various insults. Platelet count is inversely associated with MPV. Secretory granules in platelets are related to cell reactivity in hemostatic regulation. Platelets with higher MPV may have more granules and larger surface area, and this is associated with their activation. Also, platelets express Toll-like receptors (TLRs) so they recognize various molecular patterns in microorganisms. This leads to platelet activation. MPV/PC has recently been investigated as a promising predictor of mortality in critically ill sepsis patients. One such study included 120 sepsis patients, and clinical outcome was 28-day mortality [6]. Authors reported that higher MPV/PC ratio, specifically >3.71, on admission was a significant risk factor for 28-day mortality (AUC 0.81; *p* = 0.001). In their study, MPV or PC alone did not predict mortality; however, MPV/PC did and was an independent predictor of 28-day mortality. In comparison, our data showed that nonsurvivors had statistically significantly lower PC and higher MPV and MPV/PC. Clinical accuracy of MPV/PC was similar, this ratio was a good predictor of lethal outcome (similar cut-off value of 3.80, AUC 0.68; *p* ≤ 0.001). But, unlike that study, our data showed that both PC and MPV were good predictors of outcome (AUCs 0.68 and 0.63, resp.; *p* < 0.010 in both) in the peritonitis subgroup. Same was true for the pancreatitis group with even better clinical accuracy (PC: AUC 0.69, *p* = 0.006; MPV: AUC 0.79, *p* ≤ 0.001; MPV/PC: AUC 0.71, *p* = 0.003). This was confirmed by univariate odds ratios for predicting lethal outcome in the whole cohort of critically ill and injured patients. All three variables had high statistical significance. PC was an independent predictor of lethal outcome by multivariate logistic regression analysis in the whole cohort as well as MPV in the pancreatitis subgroup. So, in our study, the MPV/PC ratio was not superior to MPV or PC alone. In accordance with our results are those reported by Gao and coauthors [25]. In their investigation, a total of 124 septic shock patients were enrolled and they showed that, among platelet indices, MPV had the highest AUC of 0.81 with similar cut-off value as in our study.

Several studies investigated prognostic value of MPV regarding outcome in critically ill sepsis patients with contradictory results. In accordance with our data are results from investigation conducted by Kim and coauthors, who reported that change in MPV between hospital admission and 72 hours was an independent predictor of 28-day mortality in 345 critically ill patients. They also found that baseline MPV is a good predictor of lethal outcome with AUC of 0.65. Conclusion was that an increase in MPV during the first 72 hours of hospitalization is an independent risk factor for adverse clinical outcome in patients with severe sepsis and/or septic shock [26]. In contrast, while Zampieri and coauthors reported that an increase in MPV after admission to an ICU is independently associated with higher hospital mortality in 84 critically ill patients [27], in their study, there was no statistically significant difference between survivors and nonsurvivors regarding baseline MPV at admission. Opposite to our results, Sadaka and coauthors reported that there was no relation between MPV on day 1 of septic shock and mortality with AUC of 0.5 in large retrospective analysis. There was no statistically significant difference between survivors and nonsurvivors regarding MPV on day 1 [28]. Effect of severe sepsis on platelet count and their indices was the focus of investigation reported by Guclu and coauthors. They enrolled 145 patients with sepsis and 143 patients as control group. PC and MPV were compared between sepsis survivors and nonsurvivors. PC was only marginally different; MPV was not statistically different. But, regarding power of discrimination between septic patients and controls, MPV had very good clinical accuracy in predicting sepsis with AUC of 0.75. It was confirmed by multivariate logistic regression model in which MPV was an independent predictor of sepsis, so it can be used as diagnostic tool [29]. Statistically different MPV and MPV/PC values were found between sepsis patients and controls in another study where authors did not perform univariate and multivariate logistic regression analysis [30].

The immunoinflammatory response in critically ill septic patients is very complex with fundamental differences in the host immune response to Gram-positive bacterial pathogens compared with Gram-negative microorganisms [31–40]. In our previous study [41], we also demonstrated significant difference in cytokine profile in severe Gram-positive and Gram-negative abdominal sepsis. Therefore, our secondary endpoint in this investigation was to assess possible differences of cell ratios regarding different blood cultures in patients with documented bacteremia given that these ratios are comprised of immunocompetent cells exhibiting different behavior in different bacterial settings. When analyzing baseline and laboratory characteristics of all patients according to blood cultures, our data showed that both lymphocyte and monocyte counts were significantly higher in patients with Gram-positive blood culture compared to those with polymicrobial and negative blood cultures. Also, patients with Gram-positive blood culture had significantly lower MPV/PC ratio compared to patients with Gram-negative and polymicrobial blood cultures. When comparing polymicrobial and negative blood cultures, we found significantly higher MPV and MPV/PC values as well as significantly lower platelet count in patients with polymicrobial blood culture. Patients with Gram-negative, polymicrobial, and negative blood cultures had significantly higher PLR and MLR values in comparison with those who had Gram-positive blood culture. When comparing polymicrobial and negative blood culture, we found significantly lower PLR and MLR values in patients with polymicrobial blood culture. So, the highest levels of MLR and PLR had been found in patients with negative blood culture and lowest in patients with Gram-positive blood culture. NLR values did not differ significantly between these four subgroups of patients according to blood culture. As mentioned before, lymphocyte and platelet counts, MPV, MPV/PC, MLR, NLR, and PLR were good predictors of negative blood cultures either in the peritonitis subgroup or the pancreatitis subgroup; most of the investigated biomarkers had good discriminative power regarding negative blood cultures in both subgroups. Multivariate logistic regression analysis revealed that polymicrobial blood culture (compared to negative blood culture) is an independent predictor of lethal outcome in our cohort of critically ill and injured patients.

Surprisingly, in the literature available to us, we found only one study regarding infection-specific status of MPV in adults with sepsis [42]. Authors reported that MPV measurements from the first and second days were significantly lower in patients with Gram-positive bacteria than in patients infected with other microorganisms. This is in accordance with our findings that MPV was significantly lower in patients with Gram-positive blood culture. Apart from few studies evaluating specific platelet and MPV responses to different types of microorganisms in septic mostly very low birth weight neonates, we could not find any other study regarding MPV. To the best of our knowledge, our study is the first to investigate MPV/PC, NLR, MLR, and PLR in adult population of critically ill septic patients and patients with severe trauma regarding nature of bacteremia. We think that it might be clinically useful to help initiate adequate antibiotic therapy. According to guidelines, broad spectrum antibiotics are administered as soon as possible. The lack of causative pathogen identification in more than 40% of cases will make it more difficult to select appropriate antibiotics and may have deleterious effects on the survival of critically ill septic patients.

Our composite bioscore, in which four investigated ratios were combined, in each subgroup, significantly improved their prognostic performance regarding lethal outcome, all AUCs were around 0.80. Combination of biomarkers, in that regard, was investigated by other authors with similar results [43–46].

There is important question regarding specific timeline of investigated biomarker measurements during the first 24 hours and their predictive value regarding outcome in critically ill patients. In one study, several biomarkers like MPV, platelet count (PC), PDW (platelet distribution width)/PC, and MPV/PC were evaluated as predictors of lethal outcome [6]. Authors demonstrated that both baseline values and values at 24 hours of all four biomarkers were significantly different between survivors and nonsurvivors, with baseline MPV and PC being exception. At 48 and 72 hours, almost all of the investigated biomarkers lost their prognostic ability. Authors demonstrated that MPV/PC ratio in the early phase of severe sepsis (baseline and at 24 hours) was an independent predictor of 28-day mortality. In another study, baseline values of both PC and MPV were not significantly different between survivors and nonsurvivors. At 24 and 48 hours, only PC was significantly different, MPV was not; at 72 hours, both were significantly different between survivors and nonsurvivors. Authors also calculated prognostic value of difference in both biomarkers, ΔMPV_24h_ and ΔPC_24h_, and demonstrated that both have good prognostic value regarding outcome [27]. Since repetitive measurements during the first 24 hours have not been reported in majority of studies, this question remains open. When repetitive measurements do exist, difference is always a useful parameter and might have similar or higher prognostic value than a single measurement.

Temporal variation of investigated biomarkers is another important issue. We found two studies regarding NLR with repetitive measurements. In the first one, authors demonstrated that NLR both at admission and at day 5 was statistically different between septic shock survivors and nonsurvivors. They also calculated cell counts and NLR variations from day 1 to day 5; all showed statistically significant difference between two groups [11]. The other study was focused on the impact of NLR on mortality in critically ill trauma patients. Authors demonstrated that NLRs over 10.45 and 7.91 are independent predictors of in-hospital mortality at days 2 and 5, respectively [13]. Our group, being well aware of multiple measurement importance, recently published a study regarding NLR in a different setting, pediatric acute appendicitis (AA). NLR was measured in three different time points with regard to surgery: preoperative, on day 1, and day 3 postoperatively. We demonstrated that NLR provides good monitoring of progression of AA in children and that its cutoff values may help in distinguishing the phases of AA; therefore, it could be used in diagnosis of AA in pediatric population [47]. In the literature available to us, we did not find any studies regarding MLR and PLR with repetitive measurements. Several studies focused on repetitive measurements of PC and MPV and their prognostic value regarding outcome in patients with sepsis and/or septic shock. Kim and coauthors investigated whether the change in MPV between hospital admission and 72 hours (ΔMPV_72h-adm_) predicts 28-day mortality in severe sepsis and/or septic shock [26]. Authors demonstrated that the rate of MPV increase was significantly higher in nonsurvivors and that, in multivariate analysis, ΔMPV_72h-adm_ was an independent predictor of 28-day mortality. It should be noted that both baseline MPV and ΔMPV_72h-adm_ had good clinical accuracy in predicting lethal outcome, with AUCs of 0.65 and 0.69, respectively. In another study, authors investigated the impact of various platelet indices as prognostic markers of septic shock. Among them were PC and MPV, all with repetitive measurements, from baseline, first five consecutive days, penultimate, and last day of hospital stay, so there were 8 time points altogether [25]. Clinical accuracy of PC and MPV in predicting lethal outcome was very different but consistent within all time points for each biomarker. AUCs for PC were low in all time points, all below 0.5, whereas AUCs for MPV were high in all time points, ranging from 0.66 to 0.88.

Next step in our research is to analyze different subsets of lymphocytes and to explore phenotypic markers. This is the focus of our forthcoming study.

Our present study has several limitations. It is a single-center observational study, so it was difficult to avoid potential remains of residual confounding. Our subgroups were uneven, with smaller number of patients in the pancreatitis subgroup and the trauma subgroup than in the peritonitis subgroup and the trauma with secondary sepsis subgroup. Therefore, trends and patterns in investigated cell counts and ratios that we found should be validated in larger patient population, so further studies are warranted. We cannot generalize our results to other subgroups of critically ill and injured patients. Also, given the fact that the time point of measurement in our study, although in line with most of the other similar studies, might not always coincide with the worst measurement in the first 24 hours, we cannot claim that results would be the same if the worst measurement would always be used for calculation. Another limitation of our study is single measurement of investigated biomarkers.

## 5. Conclusions

This study demonstrates a clear relationship between higher NLR and MPV/PC levels and lethal outcome in critically ill patients with peritonitis and pancreatitis, whereas MLR and PLR did not differ significantly between survivors and nonsurvivors. Our data showed that there was no significant difference of investigated biomarkers between survivors and nonsurvivors in trauma patients with and without sepsis except for significantly higher PLR in trauma without sepsis nonsurvivors. Independent predictor of lethal outcome was NLR in the whole cohort and in the peritonitis subgroup as well as MPV in the pancreatitis subgroup. For the first time, we demonstrate statistically significant differences in MPV/PC, MLR, and PLR values regarding nature of bacteremia. In general, the lowest levels had been found in patients with Gram-positive blood cultures. In heterogeneous cohort subgroup, analysis is essential. Therefore, trends and patterns in investigated cell counts and ratios that we found should be validated in larger patient population, so further studies are warranted. We cannot generalize our results to other subgroups of critically ill and injured patients.

## Figures and Tables

**Table 1:** Baseline characteristics of all patients according to outcome.

Characteristics	All patients	Survivors	Nonsurvivors
Number of patients (%)	392 (100%)	213 (54.3%)	179 (45.7%)
Males, number (%)	236 (60.2%)	149 (70%)	87 (48.6%)
Age (years)	53.67 ± 18.26	45.66 ± 16.86	63.21 ± 15.02
Peritonitis	196 (50.0%)	93 (47.4%)	103 (52.6%)
Pancreatitis	67 (17.1%)	28 (41.8%)	39 (58.2%)
Trauma + sepsis	83 (21.2%)	57 (68.7%)	26 (31.3%)
Trauma	46 (11.7%)	35 (76.1%)	11 (23.9%)
Gram-positive blood cultures, number (%)	65 (16.6%)	53 (24.9%)	12 (6.7%)
Gram-negative blood cultures, number (%)	15 (3.8%)	11 (5.2%)	4 (2.2%)
Polymicrobial blood cultures, number (%)	184 (46.9%)	53 (24.9%)	131 (73.2%)
Negative blood cultures, number (%)	128 (32.7%)	96 (45.1%)	32 (17.9%)

**Table 2:** Laboratory characteristics of all patients according to outcome.

Characteristics	All patients	Survivors	Nonsurvivors	*p* value
WBC count (10^9^/L)	12.74 ± 5.59	12.91 ± 5.31	12.53 ± 5.91	*p* = 0.312
Neutrophil (10^9^/L)	10.40 (7.32–13.42)	10.30 (7.30–13.75)	10.40 (7.40–13.05)	*p* = 0.728
Lymphocyte (10^9^/L)	0.95 (0.61–1.31)	1.06 (0.77–1.45)	0.81 (0.55–1.20)	*p* ≤ 0.001^∗∗^
Monocyte (10^9^/L)	0.60 (0.35–0.92)	0.64 (0.40–1.03)	0.56 (0.29–0.80)	*p* ≤ 0.001^∗∗^
Platelet (10^9^/L)	241.63 ± 152.29	267.41 ± 156.04	210.94 ± 142.15	*p* ≤ 0.001^∗∗^
MPV (fL)	9.05 ± 1.76	8.61 ± 1.27	9.61 ± 2.11	*p* ≤ 0.001^∗∗^
MPV/PC ratio (fL 10^−5^ *μ*L^−1^)	3.7 (2.5–6.2)	3.4 (2.3–5.3)	4.7 (3.0–8.0)	*p* ≤ 0.001^∗∗^
NLR	11.00 (7.02–15.71)	9.91 (6.17–13.78)	12.26 (7.72–18.25)	*p* = 0.001^∗∗^
MLR	0.61 (0.38–1.02)	0.62 (0.37–1.02)	0.58 (0.39–1.01)	*p* = 0.240
PLR	226.53 (162.16–354.20)	228.38 (152.71–328.59)	225.11 (166.47–410.40)	*p* = 0.848

Data are shown as number (%), mean (standard deviation, SD), or median (interquartile range, IQR) as appropriate. Significant differences are marked by ^∗∗^ (*p* < 0.01).

**Table 3:** Baseline and laboratory characteristics of all patients according to blood cultures.

Characteristics	Gram-positive blood cultures	Gram-negative blood cultures	Polymicrobial blood cultures	Negative blood cultures	*p* value
Number of patients (%)	65 (16.6%)	15 (3.8%)	184 (46.9%)	128 (32.7%)	—
Males, number (%)	40 (61.5%)	11 (73.3%)	96 (52.2%)	89 (69.5%)	—
Age (years)	50.46 ± 23.81	59.33 ± 12.66	53.71 ± 17.55	54.59 ± 16.41	*p* = 0.287
Mortality (%)	18.5%	26.7%	71.2%	25.0%	—
WBC count (10^9^/L)	13.95 ± 5.63	13.03 ± 4.27	12.20 ± 5.99	12.85 ± 5.02	*p* = 0.113
Neutrophil (10^9^/L)	11.10 (8.05–14.25)	10.80 (9.09–15.40)	10.20 (6.43–13.30)	9.96 (7.41–13.40)	*p* = 0.226
Lymphocyte (10^9^/L)	1.14 (0.84–1.60)	0.92 (0.60–1.07)	0.86 (0.58–1.26)	0.97 (0.72–1.41)	*p* ≤ 0.001^∗∗^
Monocyte (10^9^/L)	0.63 (0.43–0.95)	0.48 (0.28–1.15)	0.56 (0.29–0.81)	0.67 (0.38–1.04)	*p* = 0.016^∗^
Platelet (10^9^/L)	229.02 ± 110.53	280.26 ± 167.22	215.99 ± 134.50	280.35 ± 182.70	*p* = 0.019^∗^
MPV (fL)	8.77 ± 1.49	9.11 ± 1.37	9.40 ± 1.99	8.69 ± 1.50	*p* = 0.010^∗^
MPV/PC ratio (fL 10^−5^ *μ*L^−1^)	3.5 (2.4–5.7)	5.3 (2.3–5.4)	4.4 (2.8–7.7)	3.5 (2.2–5.7)	*p* = 0.012^∗^
NLR	9.71 (5.84–12.52)	11.44 (7.37–17.41)	11.13 (7.18–17.08)	11.35 (6.71–15.39)	*p* = 0.243
MLR	0.44 (0.33–0.93)	0.54 (0.38–0.80)	0.57 (0.35–1.03)	0.67 (0.42–1.13)	*p* = 0.040^∗^
PLR	197.70 (139.53–274.90)	228.38 (191.26–376.71)	220.95 (161.51–376.52)	264.00 (168.08–378.01)	*p* = 0.010^∗^

Data are shown as number (%), mean (standard deviation, SD), or median (interquartile range, IQR) as appropriate. Significant differences are marked by ^∗^ (*p* < 0.05) or ^∗∗^ (*p* < 0.01).

**Table 4:** Baseline and laboratory characteristics of all patients according to underlying condition.

Characteristics	Peritonitis subgroup	Pancreatitis subgroup	Trauma + sepsis subgroup	Trauma subgroup	*p* value
Number of patients (%)	196 (50.0%)	67 (17.1%)	83 (21.2%)	46 (11.7%)	—
Males, number (%)	79 (40.3%)	48 (71.6%)	77 (92.8%)	32 (69.6%)	—
Age (years)	59.58 ± 17.78	54.15 ± 13.04	39.60 ± 14.86	53.20 ± 18.93	*p* ≤ 0.001^∗∗^
Mortality (%)	52.6%	58.2%	31.3%	23.9%	—
WBC count (10^9^/L)	12.83 ± 5.74	12.30 ± 4.06	12.55 ± 6.29	13.32 ± 5.64	*p* = 0.794
Neutrophil (10^9^/L)	10.55 (8.08–13.72)	10.60 (7.55–12.80)	10.00 (5.59–13.70)	10.04 (7.35–13.92)	*p* = 0.621
Lymphocyte (10^9^/L)	0.95 (0.58–1.29)	0.92 (0.69–1.28)	1.02 (0.64–1.48)	0.95 (0.58–1.28)	*p* = 0.431
Monocyte (10^9^/L)	0.54 (0.27–0.85)	0.67 (0.50–0.89)	0.61 (0.41–0.89)	0.67 (0.39–1.14)	*p* = 0.005^∗∗^
Platelet (10^9^/L)	228.56 ± 136.88	233.90 ± 123.08	245.64 ± 131.20	301.31 ± 249.50	*p* = 0.032^∗^
MPV (fL)	9.15 ± 1.68	9.67 ± 2.51	8.44 ± 0.96	8.90 ± 1.62	*p* ≤ 0.001^∗∗^
MPV/PC ratio (fL 10^−5^ *μ*L^−1^)	3.6 (2.7–6.2)	4.4 (2.2–6.6)	3.6 (2.4–5.7)	4.2 (2.0–6.5)	*p* = 0.794
NLR	11.20 (7.87–17.25)	11.35 (6.48–15.74)	9.10 (5.08–15.19)	11.13 (7.64–14.59)	*p* = 0.035^∗^
MLR	0.51 (0.34–0.94)	0.67 (0.46–1.16)	0.59 (0.33–0.94)	0.71 (0.45–1.21)	*p* = 0.002^∗∗^
PLR	227.90 (167.65–362.89)	223.28 (140.65–377.88)	210.41 (147.89–337.60)	243.24 (148.22–380.69)	*p* = 0.652

Data are shown as number (%), mean (standard deviation, SD), or median (interquartile range, IQR) as appropriate. Significant differences are marked by ^∗^ (*p* < 0.05) or ^∗∗^ (*p* < 0.01).

**Table 5:** Univariate odds ratios of variables for predicting lethal outcome in all patients.

Variables	Standard *β* value	OR	95% confidence interval		*p* value
			Lower bound	Upper bound	
Female gender	0.901	2.462	1.627	3.726	0.001^∗∗^
Age	0.064	1.066	1.050	1.081	0.001^∗∗^
Underlying condition (peritonitis)	1.260	3.524	1.693	7.336	0.001^∗∗^
Underlying condition (pancreatitis)	1.489	4.432	1.926	10.199	0.001^∗∗^
Underlying condition (trauma with secondary sepsis)	0.372	1.451	0.639	3.299	0.374
Gram-positive blood culture	−0.387	0.679	0.323	1.429	0.308
Gram-negative blood culture	0.087	1.091	0.325	3.667	0.888
Polymicrobial blood culture	2.004	7.415	4.445	12.370	0.001^∗∗^
WBC	−0.012	0.988	0.953	1.024	0.508
Neutrophil	−0.001	0.999	0.961	1.038	0.952
Lymphocyte	−0.223	0.800	0.592	1.083	0.149
Monocyte	−0.935	0.393	0.243	0.635	0.001^∗∗^
Platelet	−0.003	0.997	0.996	0.999	0.001^∗∗^
MPV	0.361	1.435	1.247	1.651	0.001^∗∗^
MPV/PC	0.069	1.072	1.030	1.114	0.001^∗∗^
NLR	0.036	1.037	1.014	1.060	0.001^∗∗^
MLR	−0.171	0.842	0.590	1.202	0.344
PLR	0.001	1.001	1.000	1.001	0.286

Significant differences are marked by ^∗∗^ (*p* < 0.01).

**Table 6:** Independent predictors of lethal outcome by multivariate logistic regression analysis in all patients.

Variables	Standard *β* value	OR	95% confidence interval		*p* value
			Lower bound	Upper bound	
Female gender	1348	3851	1712	8664	0.001^∗∗^
Age	0.115	1.122	1.089	1.155	0.001^∗∗^
Underlying condition (pancreatitis)	1.184	3.266	1.120	11.234	0.040^∗^
Polymicrobial blood culture^#^	3.739	42.074	14.174	124.893	0.001^∗∗^
Platelet	−0.004	1.004	1.001	1.007	0.017^∗^
NLR	0.049	1.051	1.013	1.089	0.007^∗∗^

^#^Polymicrobial blood culture compared to negative blood culture. Significant differences are marked by ^∗^ (*p* < 0.05) or ^∗∗^ (*p* < 0.01).

## Data Availability

Reasonable requests for data, up to 12 months after initial publication, will be considered by corresponding author.

## Supplementary Materials

Supplementary Materials Table 1: baseline and laboratory characteristics of the peritonitis subgroup according to outcome. Table 2: clinical accuracy of baseline biomarkers in predicting lethal outcome in the peritonitis subgroup. Table 3: clinical accuracy of baseline biomarkers in predicting negative blood culture in the peritonitis subgroup. Table 4: baseline and laboratory characteristics of the pancreatitis subgroup. Table 5: clinical accuracy of baseline biomarkers in predicting lethal outcome in the pancreatitis subgroup. Table 6: clinical accuracy of baseline biomarkers in predicting negative blood culture in the pancreatitis subgroup. Table 7: baseline and laboratory characteristics of the trauma + sepsis subgroup. Table 8: clinical accuracy of baseline biomarkers in predicting polymicrobial blood culture in trauma patients with secondary sepsis. Table 9: baseline and laboratory characteristics of the trauma subgroup. Table 10: clinical accuracy of baseline biomarker PLR in predicting lethal outcome in the trauma subgroup. Table 11: Spearman rho correlations between biomarkers in all patients. Table 12: Spearman rho correlations between biomarkers in the trauma group. Table 13: Spearman rho correlations between biomarkers in survivors with Gram-positive and negative blood cultures. Table 14: Spearman rho correlations between biomarkers in nonsurvivors with Gram-positive and polymicrobial blood cultures. Table 15: univariate odds ratios of variables for predicting lethal outcome in the peritonitis subgroup. Table 16: independent predictors of lethal outcome by multivariate logistic regression analysis in the peritonitis subgroup. Table 17: univariate odds ratios of variables for predicting lethal outcome in the pancreatitis subgroup. Table 18: independent predictors of lethal outcome by multivariate logistic regression analysis in the pancreatitis subgroup. Figure 1: receiver operating characteristic (ROC) curve for composite bioscore in the peritonitis subgroup and the lethal outcome. Figure 2: receiver operating characteristic (ROC) curve for composite bioscore in the trauma with secondary sepsis subgroup and the lethal outcome. Figure 3: receiver operating characteristic (ROC) curve for composite bioscore in the trauma subgroup and the lethal outcome. Figure 4: scattergram for the trauma subgroup (survivors and nonsurvivors) of: A. MPV/PC versus MLR; B. NLR versus MLR; C. MPV/PC versus PLR; D. MLR versus PLR.
