# Supplementary material for: Neutrophil-to-Lymphocyte Ratio, Monocyte-to-Lymphocyte Ratio, Platelet-to-Lymphocyte Ratio, and Mean Platelet Volume-to-Platelet Count Ratio as Biomarkers in Critically Ill and Injured Patients: Which Ratio to Choose to Predict Outcome and Nature of Bacteremia?
Source: Mediators Inflamm. 2018 Jul 15;2018:3758068. doi: 10.1155/2018/3758068 (PMC6079471; doi:10.1155/2018/3758068)
Supplement: Supplementary Materials — Table 1: baseline and laboratory characteristics of the peritonitis subgroup according to outcome. Table 2: clinical accuracy of baseline biomarkers in predicting lethal outcome in the peritonitis subgroup. Table 3: clinical accuracy of baseline biomarkers in predicting negative blood culture in the peritonitis subgroup. Table 4: baseline and laboratory characteristics of the pancreatitis subgroup. Table 5: clinical accuracy of baseline biomarkers in predicting lethal outcome in the pancreatitis subgroup. Table 6: clinical accuracy of baseline biomarkers in predicting negative blood culture in the pancreatitis subgroup. Table 7: baseline and laboratory characteristics of the trauma + sepsis subgroup. Table 8: clinical accuracy of baseline biomarkers in predicting polymicrobial blood culture in trauma patients with secondary sepsis. Table 9: baseline and laboratory characteristics of the trauma subgroup. Table 10: clinical accuracy of baseline biomarker PLR in predicting lethal outcome in the trauma subgroup. Table 11: Spearman rho correlations between biomarkers in all patients. Table 12: Spearman rho correlations between biomarkers in the trauma group. Table 13: Spearman rho correlations between biomarkers in survivors with Gram-positive and negative blood cultures. Table 14: Spearman rho correlations between biomarkers in nonsurvivors with Gram-positive and polymicrobial blood cultures. Table 15: univariate odds ratios of variables for predicting lethal outcome in the peritonitis subgroup. Table 16: independent predictors of lethal outcome by multivariate logistic regression analysis in the peritonitis subgroup. Table 17: univariate odds ratios of variables for predicting lethal outcome in the pancreatitis subgroup. Table 18: independent predictors of lethal outcome by multivariate logistic regression analysis in the pancreatitis subgroup. Figure 1: receiver operating characteristic (ROC) curve for composite bioscore in the peritonitis subgroup and the lethal outc [file 3758068.f1.pdf]

Table 1. Baseline and laboratory characteristics of the peritonitis subgroup according to outcome

Characteristics	All patients	Survivors	Nonsurvivors	*p* value
Number of patients (%)	196 (100 %)	93 (47.4 %)	103 (52.6 %)	-
Males, number (%)	79 (40.3%)	49 (52.7)	30 (29.1)	*p*= 0.001**
Age (years)	59.58 ± 17.78	50.44 ± 18.75	67.83 ± 11.92	*p*≤ 0.001**
Gram-positive blood cultures, number (%)	43 (21.9 %)	31 (33.3)	12 (11.7)	*p*≤ 0.001**
Gram-negative blood cultures, number (%)	15 (7.7 %)	11 (11.8)	4 (3.9)	*p*= 0.045*
Polymicrobial blood cultures, number (%)	91 (46.4 %)	18 (19.4)	73 (70.9)	*p*≤ 0.001**
Negative blood culture number (%)	47 (24.0 %)	33 (35.5)	14 (13.6)	*p*= 0.001**
WBC count (10^9/L)	12.83 ± 5.74	13.24 ± 4.39	12.45 ± 6.73	*p*= 0.324
Neutrophil (10^9/L)	10.55 (8.08 –13.72)	11.05 (8.98 –13.77)	10.08 (6.72 –13.42)	*p*= 0.136
Lymphocyte (10^9/L)	0.95 (0.58–1.29)	1.04 (0.79–1.41)	0.76 (0.45–1.21)	*p*= 0.001**
Monocyte (10^9/L)	0.54 (0.27–0.85)	0.59 (0.36–0.98)	0.48 (0.20–0.79)	*p*= 0.008**
Platelet (10^9/L)	228.56 ± 136.88	271.78 ± 134.98	189.54 ± 127.06	*p*≤ 0.001**
MPV (fL)	9.15 ± 1.68	8.75 ± 1.47	9.55 ± 1.79	*p*= 0.001**
MPV/PC ratio (fL 10^{-5} μL^{-1})	3.6 (2.7–6.2)	3.4 (2.3–4.8)	4.5 (3.2–8.5)	*p*≤ 0.001**
NLR	11.20 (7.87–17.25)	10.31 (7.97–13.58)	12.80 (7.75–20.01)	*p*= 0.027*
MLR	0.51 (0.34–0.94)	0.51 (0.35–0.97)	0.51 (0.32–0.94)	*p*= 0.398
PLR	227.90 (167.65–362.89)	229.66 (170.27–331.68)	226.51 (166.61–416.67)	*p*= 0.289

Data are shown as number (%), mean (standard deviation, SD), or median (interquartile range, IQR) as appropriate.
Significant differences are marked by * (p < 0.05) or ** (p<0.01).

Table 2. Clinical accuracy of baseline biomarkers in predicting lethal outcome in peritonitis subgroup

Biomarker	AUC ROC	*p* value	95% Confidence Interval		Cut-off value	Sensitivity (%)	Specificity (%)	Youden index
			Lower Bound	Upper Bound				
Lymphocyte	0.639	0.001**	0.562	0.717	0.641	47.0	83.0	0.29
Monocyte	0.610	0.008**	0.531	0.688	0.287	38.8	83.9	0.22
Platelet	0.686	0.000**	0.612	0.760	200.50	58.0	74.0	0.32
MPV	0.631	0.002**	0.550	0.712	9.15	55.6	68.9	0.24
MPV/PC	0.681	0.000**	0.604	0.758	3.80	63.0	71.0	0.34
NLR	0.592	0.027*	0.512	0.672	13.25	49.0	74.0	0.23

Significant differences are marked by * (p < 0.05) or ** (p<0.01).

Table 3. Clinical accuracy of baseline biomarkers in predicting negative blood culture in peritonitis subgroup

Biomarker	AUC ROC	p value	95% Confidence Interval		Cut-off value	Sensitivity (%)	Specificity (%)	Youden index
			Lower Bound	Upper Bound				
Platelet	0.638	0.004**	0.546	0.730	234.5	63.8	60.4	0.24
MPV	0.643	0.004**	0.556	0.729	9.15	80.4	51.5	0.32
MPV/PC	0.636	0.006**	0.541	0.732	3.75	71.7	56.7	0.28
MLR	0.586	0.046*	0.489	0.683	0.6155	55.3	63.8	0.19
PLR	0.613	0.020*	0.523	0.702	241.05	61.7	62.4	0.24

Significant differences are marked by * ($p < 0.05$) or ** ($p<0.01$).

Table 4. Baseline and laboratory characteristics of the pancreatitis subgroup

Characteristics	All patients	Survivors	Nonsurvivors	*p* value
Number of patients (%)	67 (100 %)	28 (41.8 %)	39 (58.2 %)	-
Males, number (%)	48 (71.6 %)	28 (100.0 %)	20 (51.3 %)	p= 0.001**
Age (years)	54.15 ± 13.048	50.46 ± 11.79	56.79 ± 13.40	p= 0.049*
Polymicrobial blood cultures, number (%)	32 (47.8%)	0 (0.0)	32 (82.1)	$p\leq$ 0.001**
Negative blood culture number (%)	35 (52.2%)	28 (100)	7 (17.9)	$p\leq$ 0.001**
WBC count (10^9/L)	12.30 ± 4.06	11.17 ± 4.73	13.12 ± 3.32	p= 0.037*
Neutrophil (10^9/L)	10.60 (7.55 –12.80)	7.84 (5.98–12.00)	11.70 (9.34–13.20)	p= 0.005**
Lymphocyte (10^9/L)	0.92 (0.69–1.28)	1.10 (0.91–1.46)	0.84 (0.61–1.15)	p= 0.005**
Monocyte (10^9/L)	0.67 (0.50–0.89)	0.74 (0.49–1.04)	0.64 (0.50–0.86)	p= 0.162
Platelet (10^9/L)	233.90 ± 123.08	280.14 ± 107.66	200.70 ± 123.98	p= 0.008**
MPV (fL)	9.67 ± 2.51	8.40 ± 1.52	10.59 ± 2.70	$p\leq$ 0.001**
MPV/PC ratio (fL 10^{-5} μL^{-1})	4.4 (2.2–6.6)	2.8 (2.2–4.1)	5.6 (3.3–8.5)	p= 0.003**
NLR	11.35 (6.48–15.74)	6.57 (4.84–11.35)	14.29 (8.31–17.20)	$p\leq$ 0.001**
MLR	0.67 (0.46–1.16)	0.63 (0.40–1.15)	0.76 (0.53–1.16)	p= 0.360
PLR	223.28 (140.65–377.88)	276.82 (133.19–341.90)	203.47 (141.53–416.35)	p= 0.387

Data are shown as number (%), mean (standard deviation, SD), or median (interquartile range, IQR) as appropriate.
Significant differences are marked by * ($p < 0.05$) or ** ($p<0.01$).

Table 5. Clinical accuracy of baseline biomarkers in predicting lethal outcome in pancreatitis subgroup

Biomarker	AUC ROC	*p* value	95% Confidence Interval		Cut-off value	Sensitivity (%)	Specificity (%)	Youden index
			Lower Bound	Upper Bound				
Lymphocyte	0.702	0.005**	0.576	0.828	0.915	62.0	75.0	0.37
Platelet	0.698	0.006**	0.571	0.824	201.50	67.0	75.0	0.42
MPV	0.798	0.000**	0.685	0.912	8.75	73.0	80.1	0.54
MPV/PC	0.717	0.003**	0.589	0.845	4.30	71.0	78.0	0.49
NLR	0.753	0.000**	0.633	0.872	10.44	72.0	75.0	0.47

Significant differences are marked by * ($p < 0.05$) or ** ($p<0.01$).

Table 6. Clinical accuracy of baseline biomarkers in predicting negative blood culture in pancreatitis subgroup

Biomarker	AUC ROC	*p* value	95% Confidence Interval		Cut-off value	Sensitivity (%)	Specificity (%)	Youden index
			Lower Bound	Upper Bound				
Lymphocyte	0.683	0.008**	0.553	0.812	0.74	88.6	43.7	0.32
Platelet	0.630	0.047*	0.489	0.772	119.0	100	31.2	0.31
MPV	0.790	0.000**	0.678	0.901	8.95	78.8	74.2	0.53
MPV/PC	0.696	0.007**	0.556	0.835	5.29	78.8	64.5	0.43
NLR	0.721	0.002**	0.598	0.845	13.00	77.1	59.4	0.37

Significant differences are marked by * ($p < 0.05$) or ** ($p<0.01$).

Table 7. Baseline and laboratory characteristics of the trauma + sepsis subgroup

Characteristics	All patients	Survivors	Nonsurvivors	*p* value
Number of patients (%)	83 (100%)	57 (68.7%)	26 (31.3%)	-
Males, number (%)	77 (92.8%)	51 (89.5)	26 (100.0)	*p*= 0.170
Age (years)	39.60 ± 14.86	35.07 ± 10.26	49.54 ± 18.41	*p*= 0.001**
Gram-positive blood cultures, number (%)	22 (26.5%)	22 (38.6)	0 (0.0)	*p*= 0.001**
Polymicrobial blood culture number (%)	61 (73.5%)	35 (61.4)	26 (100.0)	*p*= 0.001**
WBC count (10^9/L)	12.55 ± 6.29	12.62 ± 6.51	12.41 ± 5.88	*p*= 0.891
Neutrophil (10^9/L)	10.00 (5.59–13.70)	10.00 (5.37–14.40)	9.89 (6.25–12.87)	*p*= 0.973
Lymphocyte (10^9/L)	1.02 (0.64–1.48)	1.14 (0.71–1.46)	0.76 (0.60–2.22)	*p*= 0.489
Monocyte (10^9/L)	0.61 (0.41–0.89)	0.63 (0.43–0.92)	0.56 (0.38–0.78)	*p*= 0.253
Platelet (10^9/L)	245.64 ± 131.20	239.67 ± 114.57	258.72 ± 163.70	*p*= 0.595
MPV (fL)	8.44 ± 0.96	8.36 ± 0.80	8.60 ± 1.24	*p*= 0.389
MPV/PC ratio (fL 10^{-5} μL^{-1})	3.6 (2.4–5.7)	3.3 (2.5–5.4)	4.6 (1.6–6.9)	*p*= 0.363
NLR	9.10 (5.08–15.19)	9.60 (4.97–13.74)	8.50 (5.36–18.32)	*p*= 0.883
MLR	0.59 (0.33–0.94)	0.59 (0.33–1.06)	0.60 (0.37–0.83)	*p*= 0.556
PLR	210.41 (147.89–337.60)	198.60 (145.80–336.45)	246.08 (162.22–373.41)	*p*= 0.356

Data are shown as number (%), mean (standard deviation, SD), or median (interquartile range, IQR) as appropriate.
Significant differences are marked by * ($p < 0.05$) or ** ($p<0.01$).

Table 8. Clinical accuracy of baseline biomarkers in predicting polymicrobial blood culture in trauma patients with secondary sepsis

Biomarker	AUC ROC	*p* value	95% Confidence Interval		Cut-off value	Sensitivity (%)	Specificity (%)	Youden index
			Lower Bound	Upper Bound				
Lymphocyte	0.639	0.045*	0.502	0.776	0.93	54.1	77.3	0.31
NLR	0.659	0.027*	0.532	0.787	12.97	39.3	90.9	0.30

Significant differences are marked by * ($p < 0.05$) or ** ($p<0.01$).

Table 9. Baseline and laboratory characteristics of the trauma subgroup

Characteristics	All patients	Survivors	Nonsurvivors	*p* value
Number of patients (%)	46 (100%)	35 (76.1%)	11 (23.9%)	-
Males, number (%)	32 (69.6 %)	21 (60.0 %)	11 (100.0 %)	*p*= 0.020*
Age (years)	53.20 ± 18.93	46.34 ± 16.34	75.00 ± 4.05	*p*≤ 0.001**
WBC count (10^9/L)	13.32 ± 5.64	13.88 ± 5.67	11.53 ± 5.41	*p*= 0.232
Neutrophil (10^9/L)	10.04 (7.35–13.92)	10.50 (7.66–14.30)	8.68 (5.97–11.70)	*p*= 0.212
Lymphocyte (10^9/L)	0.95 (0.58–1.28)	1.06 (0.56–1.53)	0.93 (0.77–0.96)	*p*= 0.193
Monocyte (10^9/L)	0.67 (0.39–1.14)	0.87 (0.38–1.35)	0.46 (0.39–1.05)	*p*= 0.201
Platelet (10^9/L)	301.31 ± 249.50	290.82 ± 263.91	334.70 ± 204.22	*p*= 0.616
MPV (fL)	8.90 ± 1.62	8.81 ± 1.06	9.18 ± 2.82	*p*= 0.683
MPV/PC ratio (fL 10^{-5} μL^{-1})	4.2 (2.0–6.5)	4.6 (2.9–6.6)	2.3 (1.4–6.5)	*p*= 0.415
NLR	11.13 (7.64–14.59)	11.51 (7.65–15.08)	10.26 (7.61–12.51)	*p*= 0.722
MLR	0.71 (0.45–1.21)	0.80 (0.51–1.25)	0.48 (0.43–1.10)	*p*= 0.261
PLR	243.24 (148.22–380.69)	220.33 (143.39–315.17)	378.19 (237.35–574.38)	*p*= 0.029*

Data are shown as number (%), mean (standard deviation, SD), or median (interquartile range, IQR) as appropriate.
Significant differences are marked by * ($p < 0.05$) or ** ($p<0.01$).

Table 10. Clinical accuracy of baseline biomarker PLR in predicting lethal outcome in trauma subgroup

Biomarker	AUC ROC	*p* value	95% Confidence Interval		Cut-off value	Sensitivity (%)	Specificity (%)	Youden index
			Lower Bound	Upper Bound				
PLR	0.719	0.030*	0.532	0.907	327.72	64.0	80.0	0.44

Significant differences are marked by * ($p < 0.05$) or ** ($p<0.01$).

Figure 1. A Receiver operating characteristic (ROC) curve for composite bioscore in peritonitis subgroup and the lethal outcome (AUC =0.718)

B Percentage of nonsurvivors according to the each bioscore point value in peritonitis subgroup (darkest shade – cut-off bioscore point value)

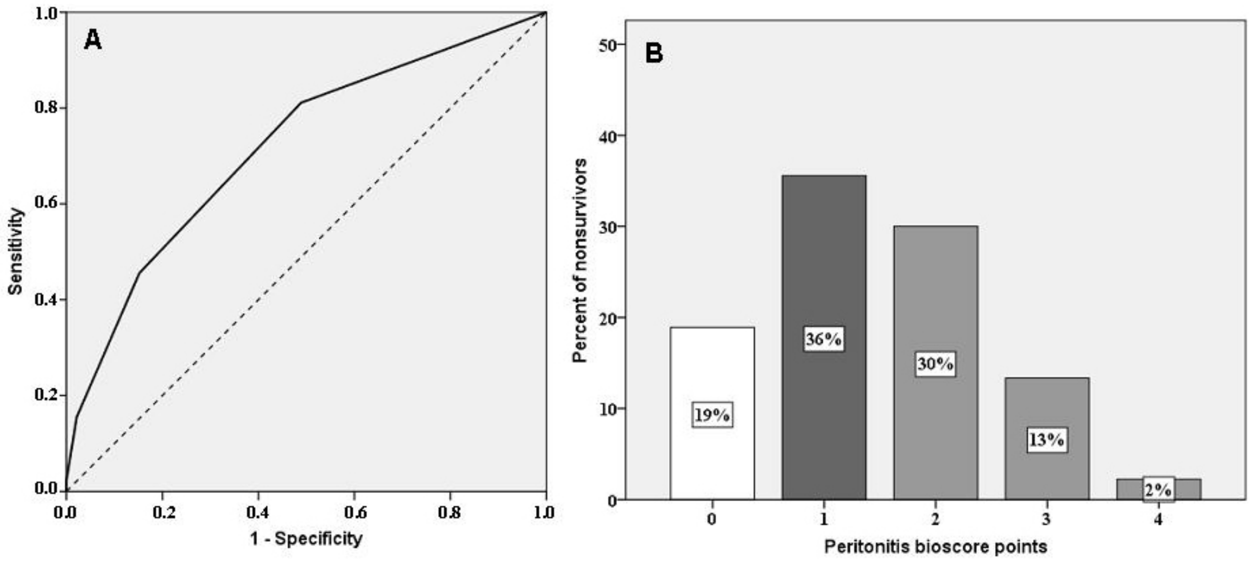

Figure 2. A Receiver operating characteristic (ROC) curve for composite bioscore in trauma with secondary sepsis subgroup and the lethal outcome (AUC = 0.782)
B Percentage of nonsurvivors according to the each bioscore point value in trauma with secondary sepsis subgroup (darkest shade – cut-off bioscore point value)

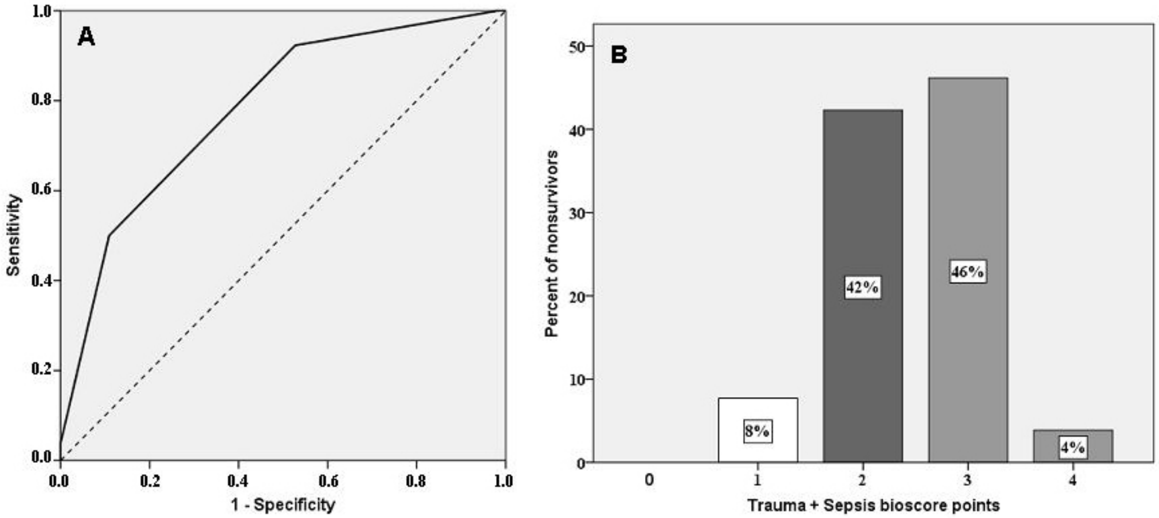

Figure 3. A Receiver operating characteristic (ROC) curve for composite bioscore in trauma subgroup and the lethal outcome (AUC = 0.823)

B Percentage of nonsurvivors according to the each bioscore point value in trauma subgroup (darkest shade – cut-off bioscore point value)

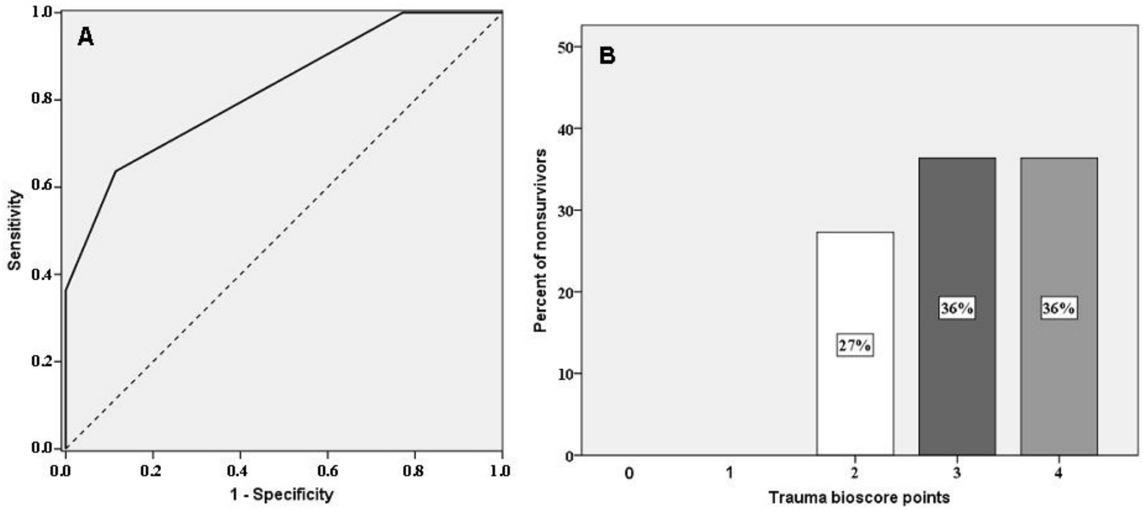

Table 11. Spearman rho correlations between biomarkers in all patients *negative correlation

**Biomarkers**	**NLR**	**MLR**	**PLR**
**MPV/PC**	**survivors**<br>0.113; p = 0.103<br>**nonsurvivors**<br>0.111; p = 0.157	**survivors**<br>-0.017; p = 0.803*<br>**nonsurvivors**<br>-0.140; p = 0.073*	**survivors**<br>-0.556; **p ≤ 0.001***<br>**nonsurvivors**<br>-0.490; **p ≤ 0.001***
**NLR**		**survivors**<br>0.497; **p ≤ 0.001**<br>**nonsurvivors**<br>0.384; **p ≤ 0.001**	**survivors**<br>0.431; **p ≤ 0.001**<br>**nonsurvivors**<br>0.470; **p ≤ 0.001**
**MLR**			**survivors**<br>0.358; **p ≤ 0.001**<br>**nonsurvivors**<br>0.454; **p ≤ 0.001**

Table 12. Spearman rho correlations between biomarkers in trauma group *negative correlation

**Biomarkers**	**NLR**	**MLR**	**PLR**
**MPV/PC**	**survivors**<br>0.278; p = 0.106<br>**nonsurvivors**<br>0.509; p = 0.110	**survivors**<br>-0.274; p = 0.111*<br>**nonsurvivors**<br>0.782; **p = 0.004**	**survivors**<br>-0.693; **p ≤ 0.001***<br>**nonsurvivors**<br>-0.909; **p ≤ 0.001***
**NLR**		**survivors**<br>0.513; **p = 0.002**<br>**nonsurvivors**<br>0.409; p = 0.212	**survivors**<br>0.330; p = 0.053<br>**nonsurvivors**<br>-0.255; p = 0.450*
**MLR**			**survivors**<br>0.473; **p = 0.004**<br>**nonsurvivors**<br>-0.655; **p = 0.029***

Figure 4. Scattergram for trauma subgroup (survivors and nonsurvivors) of: A. MPV/PC versus MLR; B. NLR versus MLR; C. MPV/PC versus PLR; D. MLR versus PLR

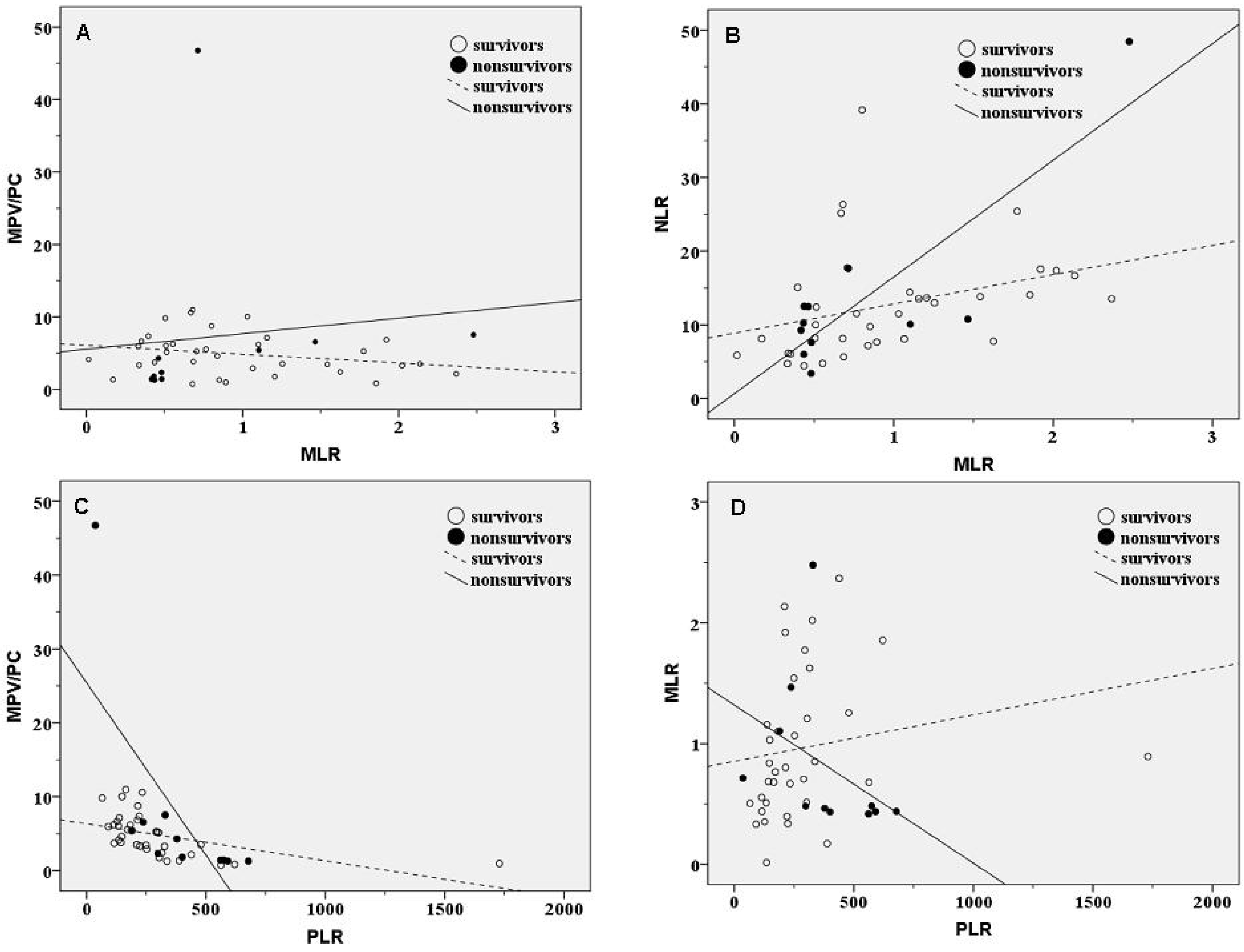

Table 13. Spearman rho correlations between biomarkers in survivors with Gram-positive and negative blood cultures *negative correlation

Biomarkers	NLR	MLR	PLR
**MPV/PC**	**Gram-positive**<br>0.072; p = 0.612<br>**negative**<br>0.069; p = 0.507	**Gram-positive**<br>0.140; p = 0.322<br>**negative**<br>- 0.086; p = 0.406	**Gram-positive**<br>-0.406; **p = 0.003***<br>**negative**<br>-0.647; **p ≤ 0.001***
**NLR**		**Gram-positive**<br>0.543; **p ≤ 0.001**<br>**negative**<br>0.669; **p ≤ 0.001**	**Gram-positive**<br>0.482; **p ≤ 0.001**<br>**negative**<br>0.481; **p ≤ 0.001**
**MLR**			**Gram-positive**<br>0.433; **p = 0.001**<br>**negative**<br>0.523; **p ≤ 0.001**

Table 14. Spearman rho correlations between biomarkers in nonsurvivors with Gram-positive and polymicrobial blood cultures *negative correlation

Biomarkers	NLR	MLR	PLR
**MPV/PC**	**Gram-positive**<br>0.050; p = 0.898<br>**polymicrobial**<br>- 0.020; p = 0.823	**Gram-positive**<br>0.317; p = 0.406<br>**polymicrobial**<br>- 0.266; **p = 0.003***	**Gram-positive**<br>0.083; p = 0.831<br>**polymicrobial**<br>-0.498; **p ≤ 0.001***
**NLR**		**Gram-positive**<br>0.748; **p = 0.005**<br>**polymicrobial**<br>0.380; **p ≤ 0.001**	**Gram-positive**<br>0.881; **p ≤ 0.001**<br>**polymicrobial**<br>0.533; **p ≤ 0.001**
**MLR**			**Gram-positive**<br>0.664; **p = 0.018**<br>**polymicrobial**<br>0.534; **p≤ 0.001**

Table 15. Univariate odds ratios of variables for predicting lethal outcome in peritonitis subgroup

Variables	Standard β value	OR	95% Confidence Interval		*p* value
			Lower Bound	Upper Bound	
Female gender	0.997	2.710	1.504	4.881	0.001**
Age	0.067	1.070	1.047	1.093	0.000**
Gram-positive blood culture	-0.092	0.912	0.366	2.275	0.844
Gram-negative blood culture	-0.154	0.857	0.233	3.158	0.817
Polymicrobial blood culture	2.258	9.560	4.251	21.499	0.000**
WBC	-0.024	0.976	0.929	1.025	0.333
Neutrophil	-0.018	0.983	0.930	1.038	0.533
Lymphocyte	-0.152	0.859	0.627	1.177	0.345
Monocyte	-0.779	0.459	0.235	0.895	0.022*
Platelet	-0.005	0.995	0.993	0.997	0.000**
MPV	0.304	1.356	1.117	1.645	0.002**
MPV/PC	0.054	1.055	1.006	1.107	0.027*
NLR	0.038	1.039	1.009	1.071	0.009**
MLR	0.008	1.008	0.615	1.652	0.974
PLR	0.000	1.000	0.999	1.001	0.758

Significant differences are marked by * ($p < 0.05$) or ** ($p<0.01$).

Table 16. Independent predictors of lethal outcome by multivariate logistic regression analysis in peritonitis subgroup

Variables	Standard β value	OR	95% Confidence Interval		*p* value
			Lower Bound	Upper Bound	
Female gender	1.753	5.769	1.918	17.358	0.002**
Age	0.113	1.120	1.077	1.165	0.000**
Polymicrobial blood culture[#]	2.774	16.019	4.225	60.741	0.000**
NLR	0.060	1.061	1.009	1.117	0.022*

[#] polymicrobial blood culture compared to negative blood culture

Significant differences are marked by * ($p < 0.05$) or ** ($p<0.01$).

Table 17. Univariate odds ratios of variables for predicting lethal outcome in pancreatitis subgroup

Variables	Standard β value	OR	95% Confidence Interval		*p* value
			Lower Bound	Upper Bound	
Age	0.039	1.040	0.999	1.082	0.040*
WBC	0.129	1.138	0.996	1.299	0.038*
Neutrophil	0.165	1.180	1.022	1.362	0.024*
Lymphocyte	-1.611	0.200	0.052	0.767	0.019*
Monocyte	-1.000	0.368	0.091	1.483	0.160
Platelet	-0.006	0.994	0.990	0.999	0.012*
MPV	0.582	1.789	1.234	2.594	0.002**
MPV/PC	0.310	1.363	1.066	1.744	0.014*
NLR	0.167	1.182	1.061	1.316	0.002**
MLR	0.454	1.574	0.538	4.608	0.408
PLR	0.000	1.000	0.997	1.003	0.999

Significant differences are marked by * ($p < 0.05$) or ** ($p<0.01$).

Table 18. Independent predictors of lethal outcome by multivariate logistic regression analysis in pancreatitis subgroup

Variables	Standard β value	OR	95% Confidence Interval		*p* value
			Lower Bound	Upper Bound	
Age	0.136	1.146	1.031	1.274	0.012*
Lymphocyte	-3.639	0.026	0.001	1.140	0.039*
MPV	1.066	2.903	1.111	7.588	0.030*

Significant differences are marked by * ($p < 0.05$) or ** ($p<0.01$).
